# Dense Disordered Jammed Packings of Hard Spherocylinders with a Low Aspect Ratio: A Characterization of Their Structure

**DOI:** 10.1021/acs.jpcb.3c03195

**Published:** 2023-07-21

**Authors:** Hugo Imaz González, Giorgio Cinacchi

**Affiliations:** † Departamento de Física Teórica de la Materia Condensada, Universidad Autónoma de Madrid, Ciudad Universitaria de Cantoblanco, E-28049 Madrid, España; ‡ Instituto de Física de la Materia Condensada (IFIMAC), Universidad Autónoma de Madrid, Ciudad Universitaria de Cantoblanco, E-28049 Madrid, España; § Instituto de Ciencias de Materiales “Nicolás Cabrera”, Universidad Autónoma de Madrid, Ciudad Universitaria de Cantoblanco, E-28049 Madrid, España

## Abstract

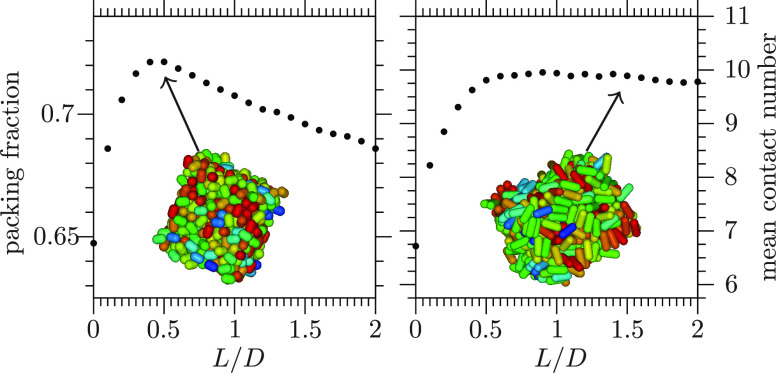

This work numerically investigates dense disordered (maximally random) jammed packings of hard spherocylinders of cylinder length *L* and diameter *D* by focusing on *L*/*D* ∈ [0,2]. It is within this interval that one expects that the packing fraction of these dense disordered jammed packings ϕ_MRJ hsc_ attains a maximum. This work confirms the form of the graph ϕ_MRJ hsc_ versus *L*/*D*: here, comparably to certain previous investigations, it is found that the maximal ϕ_MRJ hsc_ = 0.721 ± 0.001 occurs at *L*/*D* = 0.45 ± 0.05. Furthermore, this work meticulously characterizes the structure of these dense disordered jammed packings via the special pair-correlation function of the interparticle distance scaled by the contact distance and the ensuing analysis of the statistics of the hard spherocylinders in contact: here, distinctly from all previous investigations, it is found that the dense disordered jammed packings of hard spherocylinders with 0.45 ≲ *L*/*D* ≤ 2 are isostatic.

## 1 Introduction

Systems of hard particles are basic model systems with which to investigate [(soft-)condensed] states of matter.^1,2^ One aspect of this investigation concerns the determination of those packings, i.e., single configurations of hard particles that satisfy the nonoverlap constraint, that are very dense; in particular, the determination of those packings that are the densest under certain conditions.^1,2^ One example is the determination of the absolutely densest packings; another example is the determination of the relatively densest packings among those that are classifiable as disordered and jammed.^1,2^

To date, most work has focused on unicomponent packings of hard (three-dimensional) spheres. Since (at least) J. Kepler,^3^ it is (essentially) known that hard spheres pack most densely in the hexagonal-close-packed crystal and its stacking variants that include the face-centered-cubic lattice. If ρ is the number density and *v* the volume of the hard particles, the maximal value of the packing fraction ϕ = ρ*v* that hard spheres attain is .^4^ More complicated and subtler is the problem of the random close packing^5^ or, more modernly and precisely, the maximally random jammed (MRJ) state^6^ of hard spheres. The difficulty in finding first a consensus definition of this nonequilibrium state and then a rigorous analytic calculation of ϕ_MRJ hs_ is palliated by the relative ease with which this nonequilibrium state and ϕ_MRJ hs_ ≃ 0.64 are reproduced in numerical and real experiments.^5−7^

Similar numerical and real experiments can be adapted to investigate the densest-known and dense disordered (maximally random) jammed packings of hard-nonspherical particles.

Numerous hard-nonspherical particles whose densest-known and dense disordered jammed packings have been investigated are actually familiar since the school years:

(i) Ellipsoids, both uniaxial and biaxial:^8−17^ Arguably, they are the most direct generalization of hard spheres since an ellipsoid is an affine transformation of a sphere; yet, the densest-known packings of hard ellipsoids are significantly more complicated and denser^8,9,11,17^ than the packings that result from applying the same affine, ϕ-invariant, transformation to the densest packings of hard spheres.
(ii) (Circular right) Cylinders: The only hard-nonspherical particle for which a mathematical proof of the densest packings was released,^18^ and an experimental measurement of the probability distribution of the number of contacts per hard particle in dense disordered jammed packings was reported.^19^
(iii) The Platonic polyhedra as well as the Archimedean polyhedra:^20−29^ Out of all of these hard polyhedra, the hard tetrahedron emerged for the assortment of its candidate densest-known packings^20−24,26^ and seemingly being the hard convex nonspherical particle that disorderedly packs most densely.^24,25,27,29^

**Figure 1:**
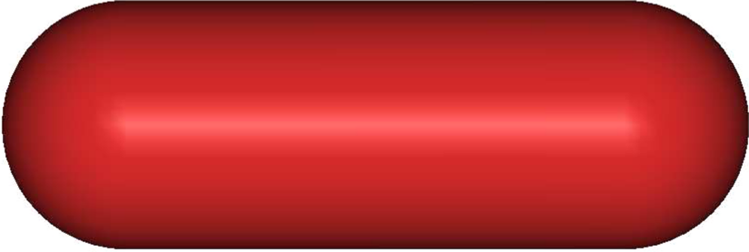
Example spherocylinder, specifically one with *L*/*D* = 2, with *L* as the length of the central cylindrical part and *D* as the diameter of the central cylindrical part and of the two extremal hemispherical parts. The image was produced by the program QMGA.^30^

One more specialist hard-nonspherical particle is the hard spherocylinder. It is formed by capping both ends of a hard cylinder of a length *L* and a diameter *D* with an equidiameter hard hemisphere (Figure 1) so that
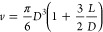


This seeming complication is instead very useful. L. Onsager did introduce this type of hard-nonspherical particles so as to show that the expression of their excluded volume is unusually very simple.^31^ This simplification facilitates any theoretical investigation of the isotropic fluid—nematic liquid-crystal phase behavior of hard-spherocylinder systems.^31−33^ To detect whether two hard spherocylinders would overlap or not amounts to computing the shortest distance between two segments, for which the algorithm is very simple. This simplification facilitates any numerical simulation investigation of hard-spherocylinder packings and systems; in particular, it facilitated the investigation of their complete phase behavior. In addition to the lower-density isotropic phase and the higher-density crystal phase, it features a plastic(rotator)-crystal phase for values of *L*/*D* sufficiently close to 0 and two liquid-crystal phases for values of *L*/*D* sufficiently far from 0.^34^ The combination of a central cylinder with two extremal hemispheres suggests to combine the results for the densest hard-sphere packings^3,4^ and those for the densest hard-cylinder packings^18^ to confidently surmise that
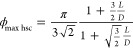
for the densest hard-spherocylinder packings.^35^

Dense disordered compact hard-spherocylinder packings have also been already investigated.^36−43^ These previous works agree that hard spherocylinders disorderedly pack more densely than hard spheres, provided the value of *L*/*D* is not too large. The values of the packing fraction that they report are, however, not concordant.^36−43^ In addition, not all of these previous works report the mean numbers of contacts per hard spherocylinder. Those that do report these mean numbers are, however, not concordant, and the values that they report are always hypostatic, even for larger values of *L*/*D.*^36,39,41,43^

Under these circumstances, this work would like to revisit dense disordered jammed packings of hard spherocylinders by focusing on *L*/*D* ∈ [0,2]. It calculates not only their packing fraction ϕ_MRJ hsc_ and the mean number of contacts per hard spherocylinder ⟨*n*_c_⟩ but also further and meticulously characterizes their microstructure. Here, this characterization is based on the special pair-correlation function *g*(*s*) of the scaled distance *s* that is obtained by dividing the distance between two hard-spherocylinder centers by their orientation-dependent contact distance.

By essentially using Monte Carlo numerical simulations in the isobaric(-isothermal) ensemble, dense packings are produced that are effectively MRJ (section 2.1). The subroutine that analyzes these jammed configurations to provide *g*(*s*) also provides a number of structural descriptors by which one could monitor how the microstructure of the MRJ packings changes as *L*/*D* increases from the hard-sphere point *L*/*D* = 0 (section 2.2).

Here, comparably to two previous works,^41,43^ it is found that the maximal ϕ_MRJ hsc_ = 0.721 ± 0.001 occurs at *L*/*D* = 0.45 ± 0.05. Concurrently to attaining this maximum, ⟨*n*_c_⟩ nearly attains the isostatic value of 2 × 5 = 10. Here, distinctly from all previous works,^36,39,41,43^ it is found that the isostatic value is maintained at larger values of *L*/*D* (section 3).

One observes that *L*/*D* ≃ 0.45 seemingly coincides with the value of *L*/*D* at which the plastic(rotator)-crystal phase disappears in the equilibrium phase diagram,^34,44^ while it still is too small a value for allowing liquid-crystal phases to appear in the equilibrium phase diagram.^34^ One may surmise that *L*/*D* ≃ 0.45 is “optimal” for all of these three reasons: (i) it is the value of *L*/*D* for which the MRJ state is the densest; (ii) it is the smallest value of *L*/*D* for which isostaticity is obtained in the MRJ state; and (iii) it is the smallest value of *L*/*D* for which neither a plastic(rotator)-crystal phase nor a liquid-crystal phase are thermodynamically stable. In the future, to assess whether the concurrence of all of these facts is a mere coincidence or rather the symptom of anything more profound in the hard-nonspherical-particle (jammed) configuration space may deserve close attention (section 4).

## 2 Methods

### 2.1 Production of the Dense Disordered Jammed Packings

To produce dense disordered jammed packings of hard spherocylinders, it was opted for a procedure that essentially consists of a progression of (precipitous) compressions. It is based on the Monte Carlo (MC) method^45,50,51^ in the isobaric(-isothermal) (NPT) ensemble^46,47,50,51^ with, importantly, a deformable container^48−50^ as well as the usual periodic boundary conditions.^45−51^ It should be equivalent to a stochastic version of the adaptable shrinking cell method that has been used to produce either densest-known or MRJ packings of a number of hard noncircular or nonspherical particles.^22,23,27,52,53^

One started by considering configurations of *N* hard spherocylinders that were produced in equilibrium MC-NPT calculations of as many hard spherocylinders with *L*/*D* = 5 at P* = *PD*^3^/(*k*_B_*T*) = 1, where *P* is the pressure, *k*_B_ is the Boltzmann constant, and *T* is the absolute temperature. For this value of dimensionless pressure, a system of hard spherocylinders with *L*/*D* = 5 equilibrates in the isotropic phase with ϕ ≃ 0.2.^34^ Configurations that were produced in these equilibrium MC-NPT calculations are devoid of any interparticle overlap. They remain eligible configurations should they be used to start numerical simulations of as many hard spherocylinders with the same value of *D* and a value of *L* < 5*D*.

One of these configurations was then used to start a first MC-NPT calculation at P* = 1 of a system of *N* hard spherocylinders with the same value of *D* and a value of *L* that corresponded to the value of *L*/*D* that was under consideration; the last configuration of this MC-NPT calculation was then used to start a second MC-NPT calculation at P* = 10; and so on; at each step of such a sequence of MC-NPT calculations, the value of P* was incremented by a factor of 10 until P* = 10^9^ was reached. Each of these ten MC-NPT calculations lasted 10^5^ MC cycles. In any of these MC-NPT calculations, an MC cycle consisted of 2*N* + 1 attempts of a change: with a probability *N*/(2*N* + 1), a random translation, of maximal length δ_t_, of the center of a randomly selected hard spherocylinder;^45−51^ with a probability *N*/(2*N* + 1), a random rotation, of maximal angle δ_r_, of the cylindrical axis of a randomly selected hard spherocylinder;^50^ and with a probability 1/(2*N* + 1), a random variation, of maximal length δ_c_, of a randomly selected element of the (e.g., upper) triangular 3 × 3 matrix that described the container.^48−50^ Random translations and random rotations were accepted if they did not cause any overlap of the hard particle with the remaining hard particles; random variations of the container were accepted if they passed the characteristic MC-NPT exponentiated test^46,47,50,51^ and they did not cause any overlap between the hard particles. In any of these MC-NPT calculations, for each of these three types of attempted change, the respective maximal amount of change was usually such that the respective probability of acceptance was around 20–30%. However, especially at the largest values of P*, the values of δ_t_, δ_r_, and δ_c_ could be as large as to determine a respective probability of acceptance of only a few percent. To assess whether an effectively jammed state had been obtained at last, each hard spherocylinder of the last configuration of the last MC-NPT calculation at P* = 10^9^ was progressively inflated while maintaining the same value of *L*/*D* until overlaps were detected; usually, *L* and *D* of a hard spherocylinder could be increased only by a factor smaller than 1 + 10^–7^. The configuration of these barely inflated hard spherocylinders remained essentially stable in an MC calculation in the canonical ensemble, with a deformable container, that was started from it and lasted another 10^5^ MC cycles; no systematic drift in the hard-spherocylinder positions and orientations could be observed. The configuration of these barely inflated hard spherocylinders was then considered as the final, effectively jammed configuration of the procedure.

For each value of *L*/*D* under consideration, this procedure was carried out first with *N* = 220 to acquire preliminary results and then with *N* = 500 to acquire more definitive results. In the last case, it was repeated seven times, each time starting with a different configuration of *N* = 500 hard spherocylinders with *L*/*D* = 5 that was obtained in those equilibrium MC-NPT calculations at P* = 1. For each value of *L*/*D* under consideration, the seven final effectively jammed configurations, each one with its own ϕ, were statistically analyzed.

### 2.2 Statistical Analysis of the Dense Disordered Jammed Packings

#### 2.2.1 Ordinary Pair-Correlation Functions

The most basic pair-correlation function is the positional pair-correlation function *g*(*r*). For a configuration of (hard) particles, it can be theoretically defined as
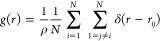
1where δ(*r*) is the usual (radial) delta function and *r*_*ij*_ = |*r⃗*_*ij*_| is the modulus of the distance vector *r⃗*_*ij*_ = *r*_*ij*_*r̂*_*ij*_ between the centroids of the *i*th (hard) particle and of the *j*th (hard) particle; while it can be practically defined as
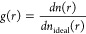
2where *dn*(*r*) is the (infinitesimal) mean number of (hard) particles that are at a distance between *r* and *r* + *dr* from a (hard) particle and *dn*_ideal_(*r*) is the analogous (infinitesimal) mean number of ideal (overlappable) particles in an equidense system of ideal (overlappable) particles.

For a configuration of (hard) nonspherical particles, (bond-)orientational pair-correlation functions can also be defined, such as
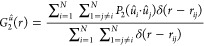
3and
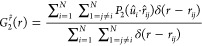
4where *P*_2_(*x*) is the second-order Legendre polynomial and *û*_*i*_ is the unit vector along a (symmetry) axis of the *i*th (hard) nonspherical particle that contributes to describing its orientation; here, *û*_*i*_ is along the cylindrical axis of the *i*th hard spherocylinder.

While *g*(*r*) informs on the positional pair correlations between the (hard) particle centroids, *G*_2_^*û*^(*r*) and *G*_2_^*r̂*^(*r*) inform on the (bond-)orientational pair correlations between the (hard) nonspherical particle (symmetry) axes and interparticle distance vectors. If the graphs of these pair-correlation functions have a (dense-)fluid-like form and *g*(*r*) attains a limit value of (essentially) unity and *G*_2_^*û*^(*r*) and *G*_2_^*r̂*^(*r*) attain a limit value of (essentially) zero, the packing is considered both positionally and orientationally globally disordered.

#### 2.2.2 Special Pair-Correlation Function and Statistics of Contacts

The statistical analysis of the dense disordered jammed packings of hard spherocylinders is actually based on the special pair-correlation function *g*(*s*) of the scaled distance *s*.

For a pair of hard spherocylinders *i* and *j*, whose distance vector between their centers is *r⃗*_*ij*_ and the respective orientation is described by the unit vector *û*_*i*_ and the unit vector *û*_*j*_, the scaled distance *s* is defined as
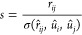
5where σ(*r̂*_*ij*_, *û*_*i*_, *û*_*j*_) is the contact distance between the hard spherocylinders *i* and *j*. It is the distance between their centers at which they contact once they are moved along the direction *r̂*_*ij*_, while their orientations *û*_*i*_ and *û*_*j*_ are maintained fixed. Since hard spherocylinders are convex: if *r*_*ij*_ ≥ σ(*r̂*_*ij*_, *û*_*i*_, *û*_*j*_) then the two hard spherocylinders do not overlap, while they do if *r*_*ij*_ < σ(*r̂*_*ij*_, *û*_*i*_, *û*_*j*_). Thus, σ(*r̂*_*ij*_, *û*_*i*_, *û*_*j*_) can be numerically determined by the simple and reliable bisection algorithm or the more sophisticated and equally reliable Brent algorithm,^54^ as the zero of the overlap function
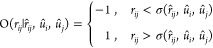
6which is a function of *r*_*ij*_ that parametrically depends on *r̂*_*ij*_, *û*_*i*_ and *û*_*j*_.

The pair-correlation function *g*(*s*) can be defined as
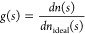
7where *dn* (*s*) is the (infinitesimal) mean number of hard particles that are at a scaled distance between *s* and *s* + *ds* from a hard particle and *dn*_ideal_ (*s*) is the analogous (infinitesimal) mean number of overlappable (ideal) particles of the same geometry in an equidense system of overlappable (ideal) particles of the same geometry. In three dimensions
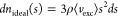
with
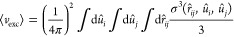
the completely orientationally averaged excluded volume,^55^ which, for two congruent hard spherocylinders, is actually equal to the following:^31^
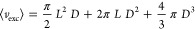
For a system of hard-nonspherical particles, the pair-correlation function *g*(*s*) offers a generalization of the usual *g*(*r*) for a system of hard spheres. It is a measure of the correlation between a pair of hard particles that are separated by the same scaled distance *s*. In particular, it considers as equally “nearby” two parallel hard spherocylinders in either an end-to-end arrangement or a side-by-side arrangement, while the ordinary pair-correlation functions would always consider the two hard spherocylinders in the former arrangement as “far apart.”

The determination of the contact distance that is required in the calculation of *g*(*s*) naturally allows for a definition of contact between two hard particles. Two hard particles are defined as exactly in contact if *s* = 1, while they can be defined as effectively in contact if 1 ≤ *s* ≤ 1 + *ds*. Thus, in a calculation of *g*(*s*), one can also tally the number of hard particles *n*_c_ that contact another hard particle and then construct the corresponding probability distribution Π(*n*_c_) from which the mean number of contacts per hard particle ⟨*n*_c_⟩ results.

Once the hard particles in contact have been detected, one can assess their degree of (bond-)orientational order. If *i* is the reference hard spherocylinder and *j* one of its *n*_c_ contacting neighbors
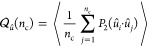
8and
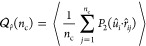
9are quantifiers of the local (bond-)orientational order of the *j* = 1, ···, *n*_c_ contacting neighbors; here, ⟨ ⟩ signify the average over all of those hard spherocylinders *i* that contact *n*_c_ hard spherocylinders *j*.

Once the hard particles in contact have been detected, one can locate the contact points. One can differentiate as to whether they occur on the cylindrical part or any of the two hemispherical parts of the two hard spherocylinders in contact. This enables the calculation of the fraction of contact points that are of the cylindrical–cylindrical type *f*_cc_, the fraction of contact points that are of the cylindrical–spherical type *f*_cs_, and the fraction of contact points that are of the spherical–spherical type *f*_ss_. One can also probe the microstructure of the contact points via the calculation of their pair-correlation function *g*_cp_(*r*), whose definition, mutatis mutandis, coincides with eqs 1 and 2.(56)

## 3 Results

By the procedure of section 2.1, dense disordered jammed packings of hard spherocylinders with *L*/*D* ∈ [0,2] have been produced; from the hard-sphere point *L*/*D* = 0, the values of *L*/*D* have been incremented in steps of 0.1; for each of these values of *L*/*D*, seven jammed configurations have been produced.

**Figure 2:**
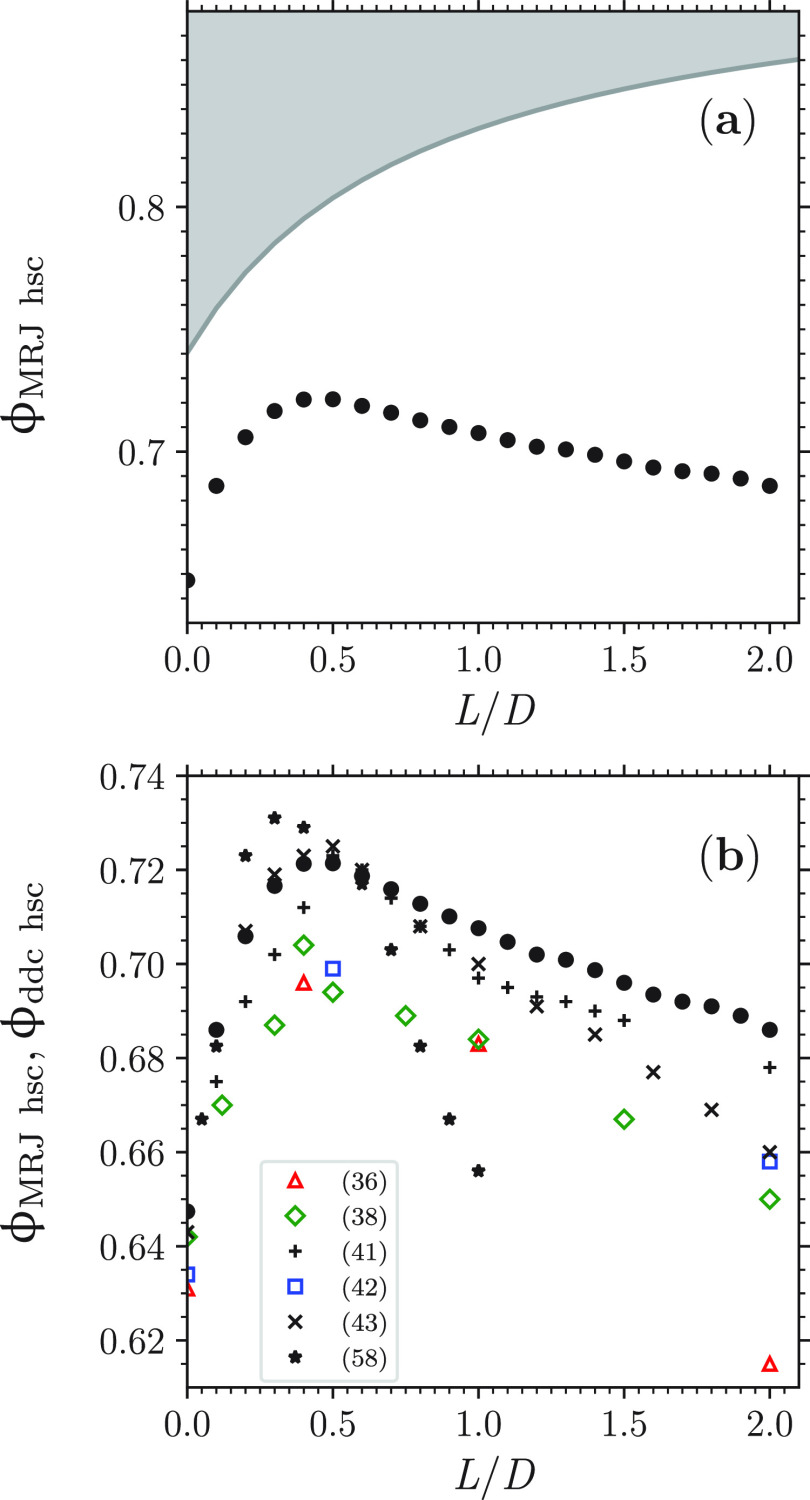
(a) Maximally random jammed state packing fraction of hard spherocylinders ϕ_MRJ hsc_ as a function of *L*/*D* (black circles; data are the average over the respective seven jammed configurations; the error bars, which typically are ∼0.001, are the corresponding standard deviation; the gray region corresponds to those values of ϕ that are prohibited as they are larger than the corresponding value of ϕ_max hsc_). (b) Comparison of the present data of ϕ_MRJ hsc_ (black circles) with previous data of ϕ of dense disordered compact packings of hard spherocylinders ϕ_ddc hsc_ (various symbols, each symbol corresponding to a previous work as the legend indicates).

For any hard-particle packing, the principal characteristic is its ϕ. For the present hard-spherocylinder MRJ packings, their ϕ_MRJ hsc_ abruptly increases as the hard-sphere point *L*/*D* = 0 is departed from, attains a maximum at *L*/*D* = 0.45 ± 0.05, whose value is 0.721 ± 0.001, and gently decreases as *L*/*D* further increases [Figure 2a]. This behavior agrees with the results of previous numerical simulations on hard-prolate-ellipsoid MRJ packings.^10,14^ Overall, this behavior also agrees with the results of previous numerical simulations on hard-spherocylinder dense disordered compact packings^36−43^ [Figure 2b]. For these previous dense disordered packings^10,14,36−43^ as in Figure 2a, the maximal ϕ occurs at a value of aspect ratio approximately equal to 3/2^10,14,36−43^ and its value is approximately equal to 0.72,^10,14,41,43^ a value significantly larger than that of ϕ_MRJ hs_.^5−7^ However, from a comparison of the present data of ϕ_MRJ hsc_ with previous data of ϕ of dense disordered compact packings of hard spherocylinders,^36,38,39,41−43^ differences are apparent: the present hard-spherocylinder MRJ packings are generally denser, especially for *L*/*D* ≳ 0.45 [Figure 2b].

The reason for these differences probably lies in the different procedures to produce dense disordered packings. Consistently with previous data of ϕ of hard-prolate-ellipsoid MRJ packings,^10,14^ the present data of ϕ_MRJ hsc_ were obtained by employing a procedure that is based on a numerical simulation method: previous hard-prolate-ellipsoid MRJ packings were produced by employing a procedure that is based on the molecular dynamics method,^10,14^ while present hard-spherocylinder MRJ packings were produced by employing a procedure that is based on the MC method. Instead, most previous data of ϕ of dense disordered compact packings of hard spherocylinders were obtained by employing a method that was termed the mechanical contraction method^36^ or its variants.^38,39,41−43^ It was already noticed^36,39,42^ that the mechanical contraction method had to be supplemented either with an MC method^36,42^ or a molecular dynamics method^39^ so as to be able to produce denser disordered packings. In the previous investigations of hard-prolate-ellipsoid MRJ packings and the present investigation of hard-spherocylinder MRJ packings, configurations were (effectively) compressed while always preserving the nonoverlap constraint and, importantly, using a deformable container and effective jamming was checked for. In the previous investigations of dense disordered compact packings of hard spherocylinders that employed the mechanical contraction method or its variants, configurations evolved in such a manner that spherocylinders ended up overlapping and these overlaps had to be removed by moving the spherocylinders within a varying container which, however, was always maintained cubic, and effective jamming was generally left unchecked.

**Figure 3:**
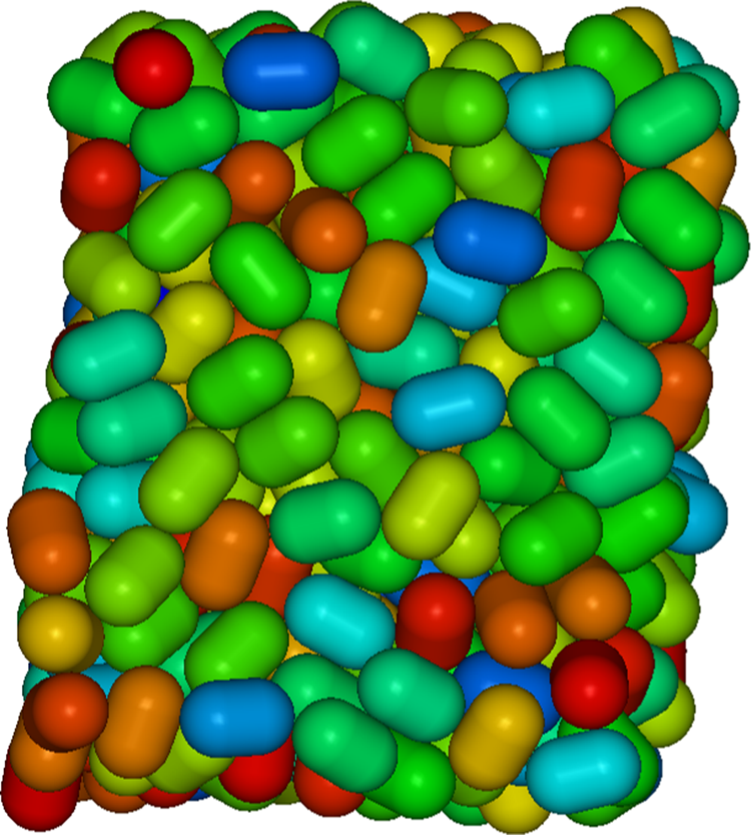
Example image of a jammed configuration of hard spherocylinders with *L*/*D* = 0.5. The color or tint of gray of a hard spherocylinder is related to the angle that its cylindrical axis forms with an arbitrary axis, e.g., the *y*-axis, of the frame of reference. The image was produced by the program QMGA.^30^

**Figure 4:**
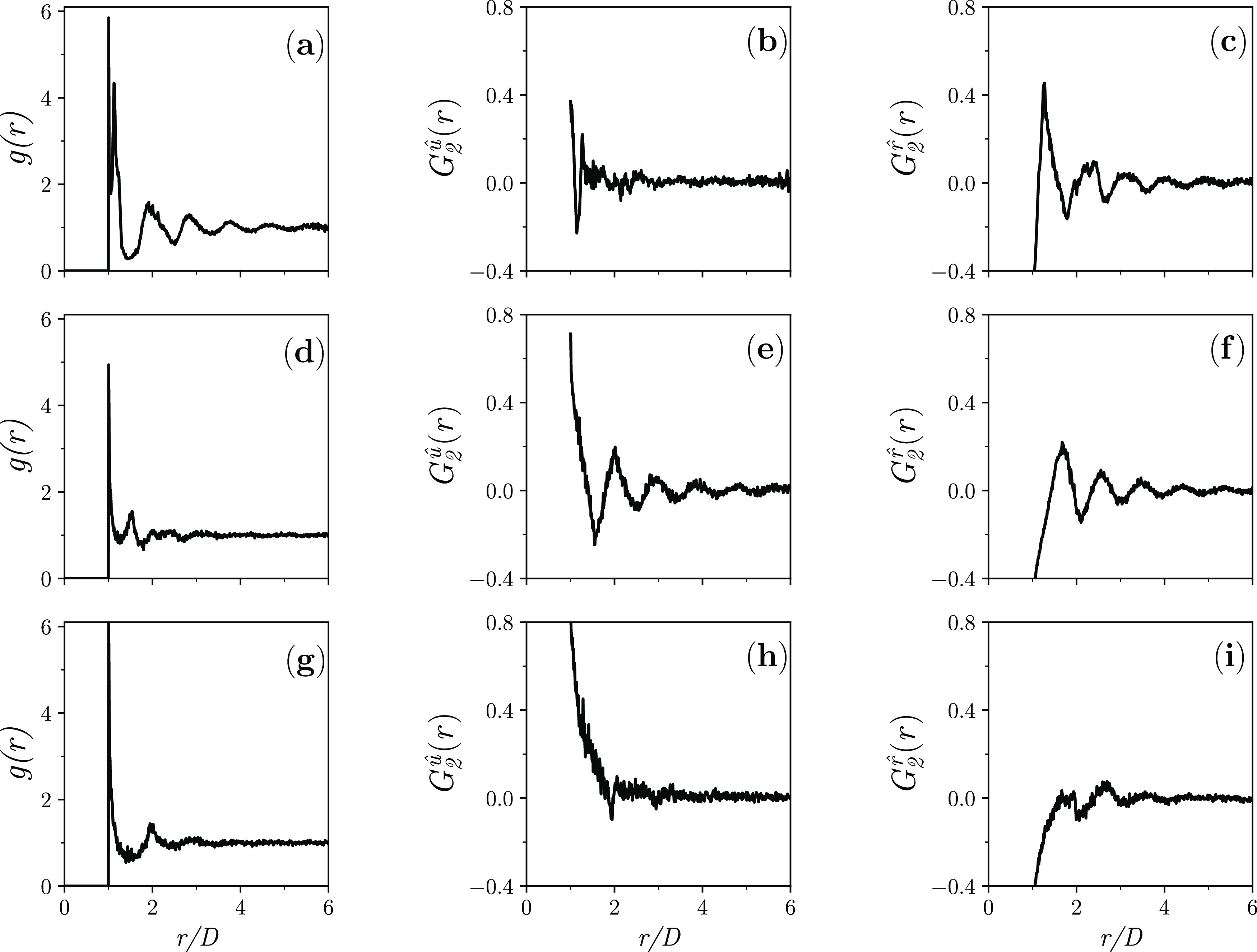
Pair-correlation functions *g*(*r*), *G*_2_^*û*^(*r*), and *G*_2_^*r̂*^(*r*) for dense disordered jammed packings of hard spherocylinders with (a, b, c) *L*/*D* = 0.3; (d, e, f) *L*/*D* = 1.1; and (g, h, i) *L*/*D* = 1.9 (in each panel, the graph is the average over the respective seven jammed configurations).

To confirm that all of the hard-spherocylinder dense jammed packings are positionally and orientationally globally disordered, the jammed configurations have been directly visualized (Figure 3), and the ordinary pair-correlation functions *g*(*r*), *G*_2_^*û*^(*r*), and *G*_2_^*r̂*^(*r*) have been calculated and observed to have a graph with a (dense-)fluid-like form and essentially attain the respective long-distance limits of unity and zero (Figure 4).

Once the positional and orientational global disorderliness of the hard-spherocylinder dense jammed packings has been confirmed, the investigation has directed to the characterization of the contacts between the hard spherocylinders.

To this aim, the principal instrument here is the pair-correlation function of the scaled interparticle distance *g*(*s*). This special pair-correlation function is not only a generalization for hard-nonspherical particle systems of the *g*(*r*) for hard-sphere systems but, when it is calculated for hard-particle jammed packings, also allows for a definition of the interparticle contacts and, consequently, their detection and the obtention of their statistics.

**Figure 5:**
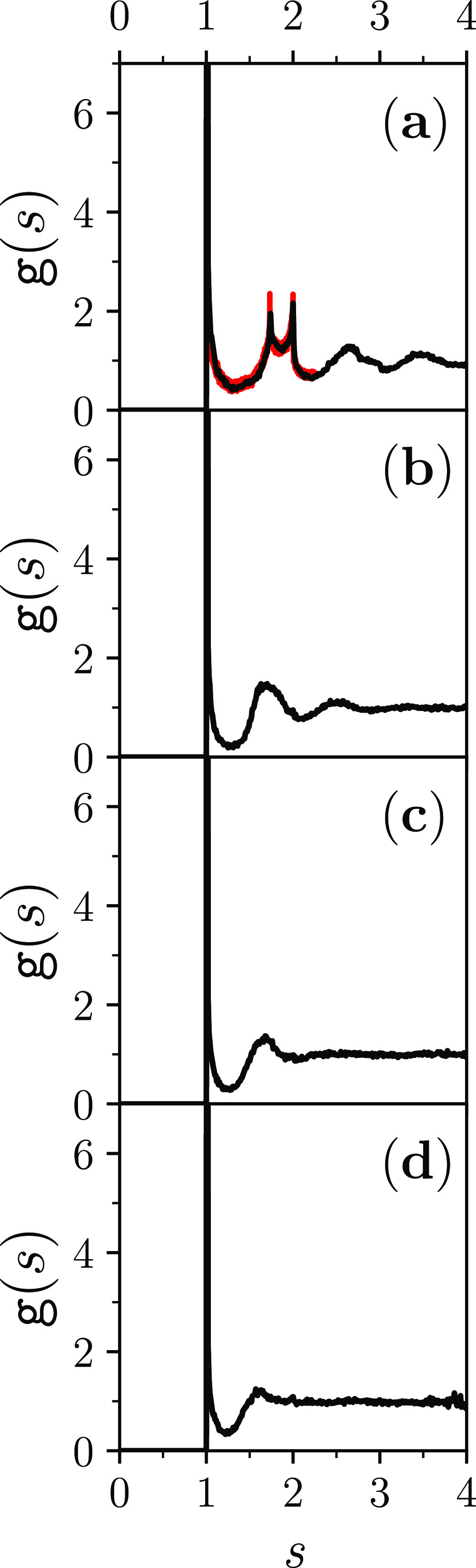
Pair-correlation function *g*(*s*) for dense disordered jammed packings of hard spherocylinders with (a) *L*/*D* = 0; (b) *L*/*D* = 0.5; (c) *L*/*D* = 1; and (d) *L*/*D* = 1.5 (in each panel, the graph is the average over the respective seven jammed configurations). In panel (a), the present *g*(*s*) (black) is compared with the accurate hard-sphere MRJ *g*(*r*/*D*) (red or gray) that was previously calculated.^57^

The (dense-)fluid-like form of the graph of *g*(*s*) and the attainment of the limit value of unity by the *g*(*s*) further confirm the positional and orientational global disorderliness of the hard-spherocylinder dense jammed packings. In particular, the (dense-)fluid-like form of the graph of *g*(*s*) excludes any discernible [plastic(rotator)-]crystallinity, while the limit value of unity that *g*(*s*) attains excludes any liquid-crystallinity (Figure 5). The changes that this special pair-correlation function experiences as the elongation of the hard spherocylinders progressively increases let one appreciate that it is the hard-sphere MRJ *g*(*r*/*D*) ≡*g*(*s*) [Figure 5a] that exhibits the most pronounced beyond-contact degree of short-range order [cf. Figure 5a with Figure 5b,c,d] and that this degree of short-range order progressively decreases as *L*/*D* increases [Figure 5b,c,d].

**Figure 6:**
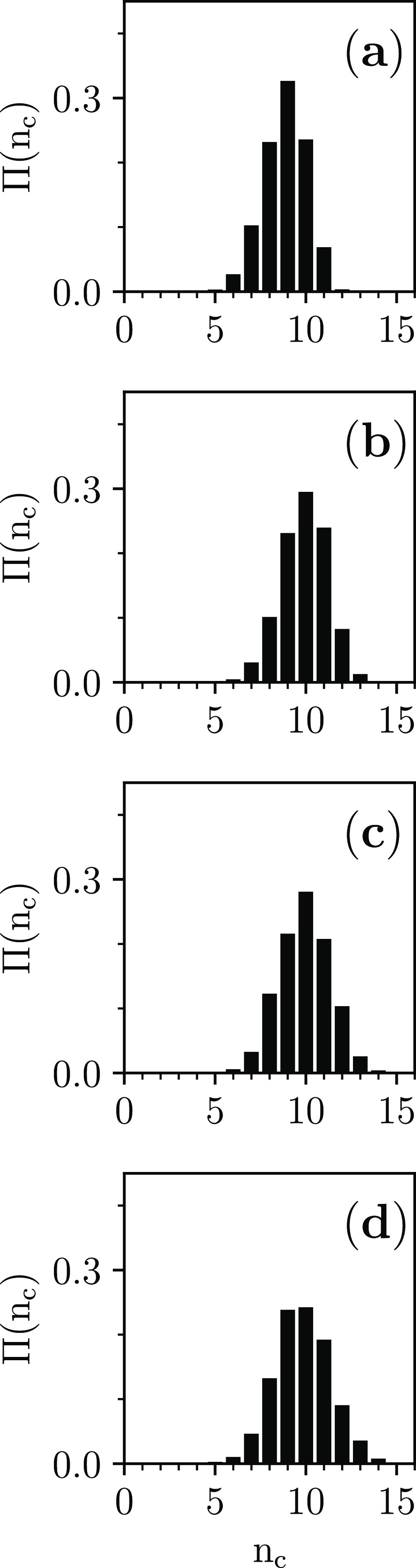
Probability distribution of the number of contacts per hard spherocylinder Π(*n*_c_) for dense disordered jammed packings of hard spherocylinders with (a) *L*/*D* = 0.2; (b) *L*/*D* = 0.7; (c) *L*/*D* = 1.2; and (d) *L*/*D* = 1.7 (in each panel, the histogram is the average over the respective seven jammed configurations).

**Figure 7:**
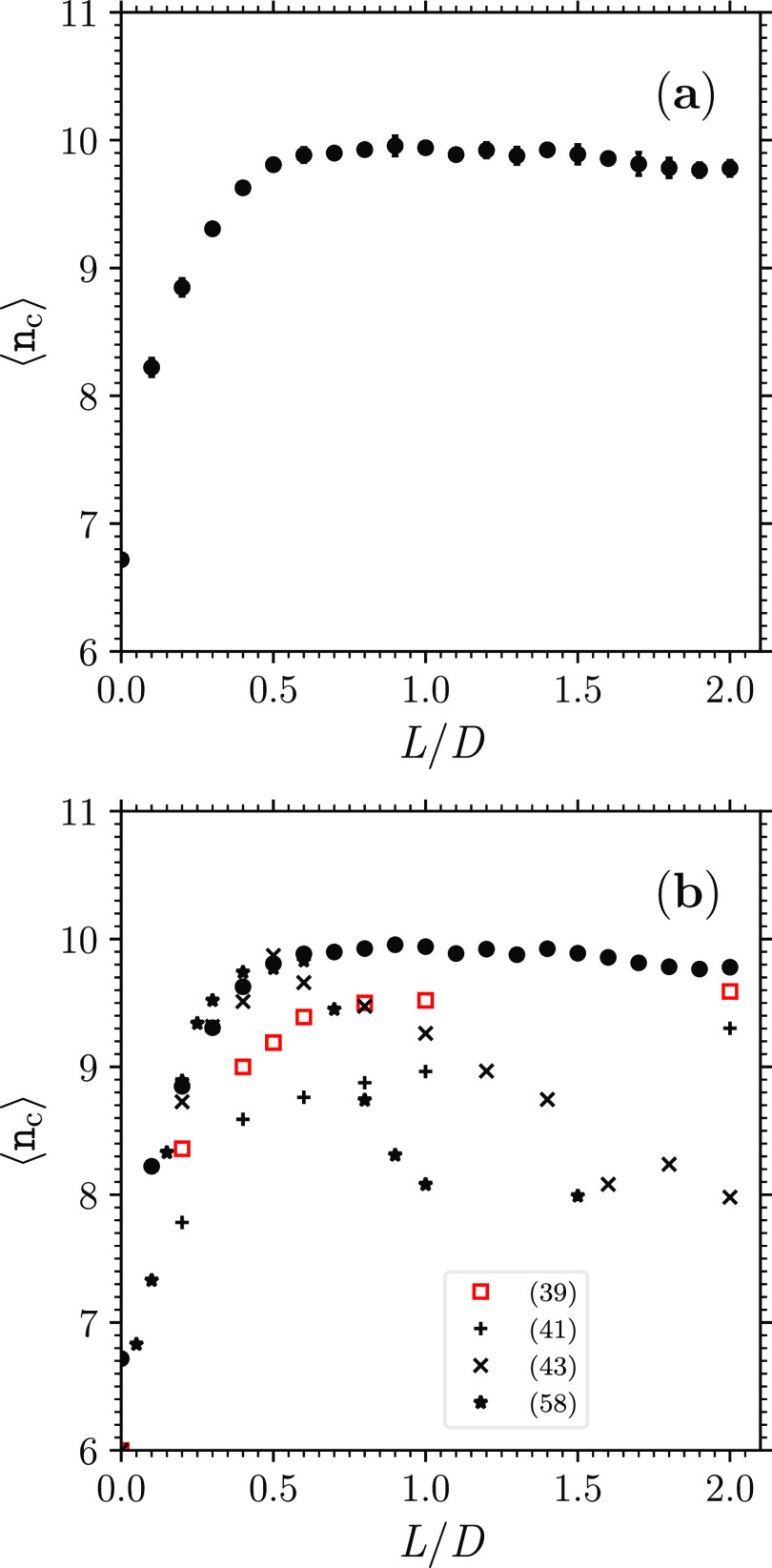
(a) Mean number of contacts per hard spherocylinder ⟨*n*_c_⟩ as a function of *L*/*D* (black circles; data are the average over the respective seven jammed configurations; the error bars, which typically are ∼0.05, are the corresponding standard deviation). (b) Comparison of the present data of ⟨*n*_c_⟩ (black circles) with previous data of ⟨*n*_c_⟩ of dense disordered compact packings of hard spherocylinders (various symbols, each symbol corresponding to a previous work as the legend indicates).

In the statistical analysis of the jammed configurations to calculate *g*(*s*), the contacts between the hard spherocylinders are detected. This allows the calculation of the probability distribution Π(*n*_c_) that a hard spherocylinder contacts with *n*_c_ neighbors (Figure 6) and, hence, of the mean number of contacts per hard spherocylinder ⟨*n*_c_⟩ [Figure 7a].

From a value ⟨*n*_c_⟩ ≃ 6.7 at the hard-sphere point *L*/*D* = 0, larger than the isostatic value 2 × 3 = 6 yet in agreement with previous real experiments on hard-sphere MRJ packings,^5,7^ ⟨*n*_c_⟩ increases with *L*/*D* [Figure 7a]. This behavior is in common with the behavior of hard-prolate-ellipsoid MRJ packings^10,14^ and of hard-spherocylinder dense disordered compact packings^36,39,41,43^ [Figure 7b].

This increase persists until the isostatic value 2 × 5 = 10 is approximately attained at a value of *L*/*D* that seemingly coincides with the value of *L*/*D* at which the maximal ϕ_MRJ hsc_ occurs [cf. Figures 2a and 7a]. The isostatic value ⟨*n*_c_⟩ ≃ 10 is maintained in the present MRJ packings of hard spherocylinders with *L*/*D* ≳ 0.45 [Figure 7a]. The maintenance of isostaticity in the present MRJ packings of hard sufficiently elongated spherocylinders is consistent with the results of previous numerical simulations on hard-prolate-ellipsoid MRJ packings, which report that ⟨*n*_c_⟩ ≃ 10 for MRJ packings of hard sufficiently elongated prolate ellipsoids.^10,14^ The maintenance of isostaticity in the present MRJ packings of hard sufficiently elongated spherocylinders is instead not consistent with the results of certain previous numerical simulations on dense disordered compact packings of hard spherocylinders, which tend to report ⟨*n*_c_⟩ < 10 also for dense disordered compact packings of hard sufficiently elongated spherocylinders,^36,39,41,43^ while other previous numerical simulations on dense disordered compact packings of hard spherocylinders did not report any value of ⟨*n*_c_⟩^37,38,40,42^ [Figure 7b].

In line with the above comments on ϕ (Figure 2), the maintenance of isostaticity in the previous MRJ packings of hard ellipsoids^10,14^ and present MRJ packings of hard spherocylinders vis-à-vis its deficiency in the previous dense disordered compact of hard spherocylinders^36,39,41,43^ (Figure 7) can be explained by the different procedures to produce dense disordered packings and their (in)capability of producing disordered packings that are very dense and jammed with a well-established contact network.

One argument has been provided to justify that the hypostatic value ⟨*n*_c_⟩ = 8 would exclusively apply to MRJ packings of hard sufficiently elongated spherocylinders.^58,59^ This argument is based on the surmise that, as *L*/*D* increases, the probability of cylinder–cylinder contacts prevails over that of cylinder–sphere contacts and that of sphere–sphere contacts so much that since the cylinder–cylinder contacts are coplanar, an effective reduction of the degrees of freedom by 1 would result; this would then lead to a mean number of contacts per hard spherocylinders equal to 2 × 4 = 8.^58,59^ Consistently with this argument, a mean-field theory predicts that, once having attained its maximum at *L*/*D* ≃ 0.5, the value of ϕ significantly drops for dense disordered jammed packings of hard spherocylinders with *L*/*D* > 0.5, and a hypostatic value ⟨*n*_c_⟩ ≃ 8 already occurs for dense disordered jammed packings of hard spherocylinders with a value of *L*/*D* as small as 1.^58,59^ These two mean-field theoretical predictions contrast with the present results in Figure 2 and in Figure 7.

This work can assess the validity of that argument and, hence, further test the validity of that mean-field theory for hard-spherocylinder dense disordered jammed packings. To this aim, one has to (1) further confirm that the hard-spherocylinder jammed packings are not only globally disordered but also locally essentially disordered; this is essential to discard the possibility that the larger values of ⟨*n*_c_⟩ that the present work finds could be due to considerable local (bond-)orientational order; (2) with this being confirmed, calculate the fraction of the three types of contacts: cylinder–cylinder *f*_cc_, cylinder–sphere *f*_sc_ and sphere–sphere *f*_ss_.

The locally (bond-)orientationally disordered character of the present hard-spherocylinder MRJ packings is confirmed by the small values that  and  take on, especially for the most probable values of *n*_c_ [Table 1a–d]. One observes that the values of  tend to be positive (nematic) for the smallest (and little probable) value of *n*_c_ and negative (antinematic) for the largest (and little probable) value of *n*_c_. These two facts agree with the common experience: if one has to positionally disorderedly arrange a number of hard rods in contact with a reference hard rod, this number has to be relatively small if the hard rods are maintained parallel to the reference hard rod, while this number can significantly increase if the hard rods are allowed to be perpendicular to the reference hard rod.

**Table 1:** Values of the Local (Bond-)Orientational Order Parameter  and  and of the Probability Π as a Function of the Number of Contacting Neighbors *n*_c_ for Dense Disordered Jammed Packings of Hard Spherocylinders with (a) *L*/*D* = 0.3; (b) *L*/*D* = 0.8; (c) *L*/*D* = 1.1; and (d) *L*/*D* = 1.8^a^

	*n*_c_			Π
(a)	3	0.1 ± 0.2	–0.1 ± 0.2	0.001 ± 0.001
	4	n.a.	n.a.	n.d.
	5	0.1 ± 0.2	–0.03 ± 0.04	0.001 ± 0.001
	6	0.03 ± 0.08	–0.11 ± 0.03	0.015 ± 0.003
	7	0.04 ± 0.03	–0.07 ± 0.01	0.06 ± 0.01
	8	0.04 ± 0.02	–0.048 ± 0.007	0.160 ± 0.009
	9	0.03 ± 0.01	–0.028 ± 0.005	0.30 ± 0.015
	10	0.021 ± 0.009	–0.007 ± 0.007	0.29 ± 0.02
	11	0.02 ± 0.015	0.009 ± 0.003	0.14 ± 0.01
	12	–0.02 ± 0.025	0.019 ± 0.006	0.024 ± 0.006
				
(b)	4	–0.002 ± 0.006	0.03 ±0.08	0.0003 ± 0.0007
	5	0.03 ± 0.06	–0.03 ± 0.08	0.0006 ± 0.0009
	6	0.05 ± 0.08	0.00 ± 0.075	0.003 ± 0.003
	7	0.09 ± 0.04	–0.03 ± 0.02	0.028 ± 0.006
	8	0.09 ± 0.03	0.001 ± 0.008	0.10 ± 0.02
	9	0.08 ± 0.01	0.02 ± 0.01	0.23 ± 0.03
	10	0.04 ± 0.01	0.038 ± 0.004	0.31 ± 0.03
	11	0.03 ± 0.01	0.044 ± 0.004	0.22 ± 0.01
	12	–0.01 ± 0.02	0.043 ± 0.009	0.08 ± 0.02
	13	–0.02 ± 0.03	0.06 ± 0.01	0.020 ± 0.006
	14	–0.05 ± 0.1	0.02 ± 0.04	0.002 ± 0.0015
	15	0.01 ± 0.02	0.01 ± 0.025	0.0003 ± 0.0007
				
(c)	3	0.03 ± 0.065	–0.1 ± 0.1	0.0003 ± 0.0007
	4	0.1 ± 0.3	–0.03 ± 0.07	0.0003 ± 0.0007
	5	0.15 ± 0.2	0.00 ± 0.09	0.0009 ± 0.001
	6	0.2 ± 0.2	–0.12 ± 0.07	0.008 ± 0.004
	7	0.18 ± 0.03	0.03 ± 0.02	0.038 ± 0.009
	8	0.19 ± 0.02	0.05 ± 0.01	0.12 ± 0.02
	9	0.14 ± 0.02	0.065 ± 0.003	0.225 ± 0.02
	10	0.10 ± 0.02	0.068 ± 0.008	0.28 ± 0.02
	11	0.06 ± 0.04	0.08 ± 0.01	0.20 ± 0.02
	12	0.00 ± 0.02	0.075 ± 0.01	0.09 ± 0.01
	13	–0.025 ± 0.04	0.08 ± 0.01	0.028 ± 0.007
	14	0.0 ± 0.1	0.08 ± 0.05	0.004 ± 0.002
	15	–0.01 ± 0.02	0.01 ± 0.01	0.0003 ± 0.0007
				
(d)	4	0.1 ± 0.2	0.0 ± 0.2	0.001 ± 0.002
	5	0.32 ± 0.15	–0.1 ± 0.1	0.002 ± 0.001
	6	0.34 ± 0.05	0.00 ± 0.02	0.015 ± 0.007
	7	0.29 ± 0.05	0.06 ± 0.04	0.055 ± 0.007
	8	0.22 ± 0.04	0.075 ± 0.009	0.137 ± 0.008
	9	0.17 ± 0.01	0.010 ± 0.008	0.22 ± 0.01
	10	0.13 ± 0.02	0.101 ± 0.007	0.25 ± 0.015
	11	0.085 ± 0.02	0.113 ± 0.009	0.18 ± 0.01
	12	0.02 ± 0.02	0.10 ± 0.01	0.091 ± 0.006
	13	0.01 ± 0.06	0.11 ± 0.03	0.034 ± 0.009
	14	–0.05 ± 0.06	0.09 ± 0.02	0.011 ± 0.006

a n.a. means “not available,” while n.d. means “not detected”.

**Figure 8:**
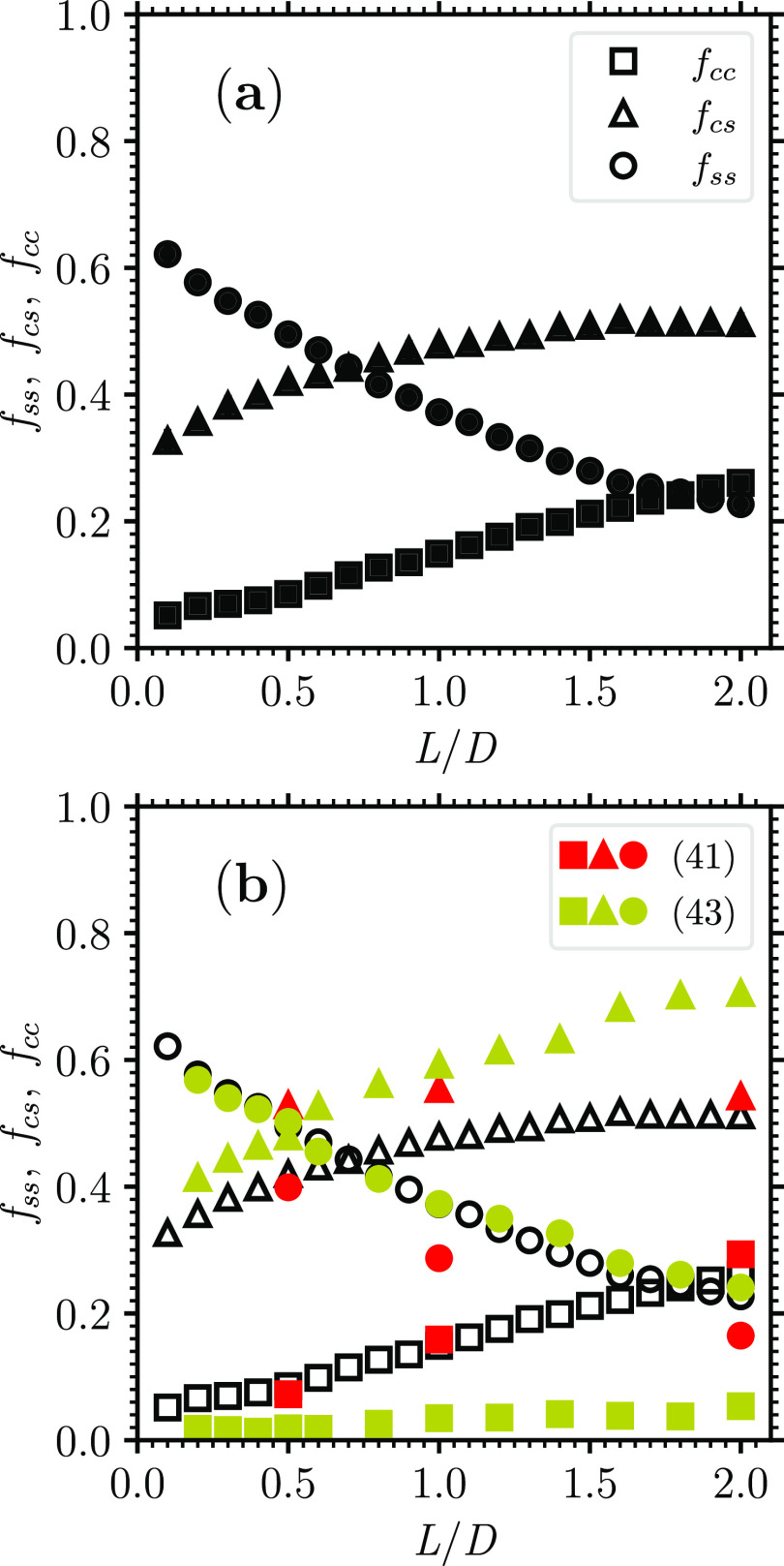
(a) Fractions of cylinder–cylinder contacts *f*_cc_, cylinder–sphere contacts *f*_cs_, and sphere–sphere contacts *f*_ss_ as a function of *L*/*D* (black empty symbols; data are the average over the respective seven jammed configurations; the error bars, which typically are ∼0.01, are the corresponding standard deviation). (One observes that *f*_cc_ and *f*_cs_ do not seem to extrapolate to zero nor *f*_ss_ to unity as *L*/*D* → 0.) (b) Comparison of the present data of *f*_cc_, *f*_cs_, and *f*_ss_ (black empty symbols) with previous data of *f*_cc_, *f*_cs_, and *f*_ss_ of dense disordered compact packings of hard spherocylinders (red or darker gray and yellow or lighter gray filled symbols, each symbol corresponding to a previous work as the legend indicates).

Expectedly, the fraction of the sphere–sphere type of contacts *f*_ss_ decreases with *L*/*D* [Figure 8a]. However, its decrease is not as marked as one could have naïvely believed; even for a value of *L*/*D* as large as 1.9, *f*_ss_ ≃ 0.22 [Figure 8a], a fraction hardly considerable as negligible. In addition, it is the fraction of the cylinder–sphere type of contacts *f*_cs_, a type of contact that still involves one of the hemispheres, that prevails for *L*/*D* ≳ 0.7 [Figure 8a]. The fraction of the cylinder–cylinder type of contacts *f*_cc_, although expectedly increasing with *L*/*D*, is never able to attain a value larger than 0.3 [Figure 8a]. In the interval *L*/*D* ∈ [0,2], at least, the role of the hemispheres in establishing contacts between the hard spherocylinders cannot be neglected.

The present results for *f*_ss_, *f*_cs_, and *f*_cc_ overall agree with previous results for these fractions^41,43^ although differences are apparent [Figure 8b]. Once they are compared with present results, the most recent previous results^43^ overestimates *f*_cs_ and even underestimates *f*_cc_ [Figure 8b]. This may actually be the cause of their reporting a decreasing hypostatic value of ⟨*n*_c_⟩ as *L*/*D* increases [Figure 7b].

The present results in Figure 8 invalidate the argument that would justify the hypostatic value ⟨*n*_c_⟩ = 8 in the case of dense disordered jammed packings of hard spherocylinders with 1 ≥ *L*/*D* ≤ 2.^58,59^ Together with the present results in Figure 2 and in Figure 7, the present results in Figure 8 refute the mean-field theory that predicts a significant drop of the value of ϕ as well as of the value of ⟨*n*_c_⟩ as *L*/*D* ≳ 0.5 so that ⟨*n*_c_⟩ ≃ 8 already for dense disordered jammed packings of hard spherocylinders with *L*/*D* > 1.^58,59^

**Figure 9:**
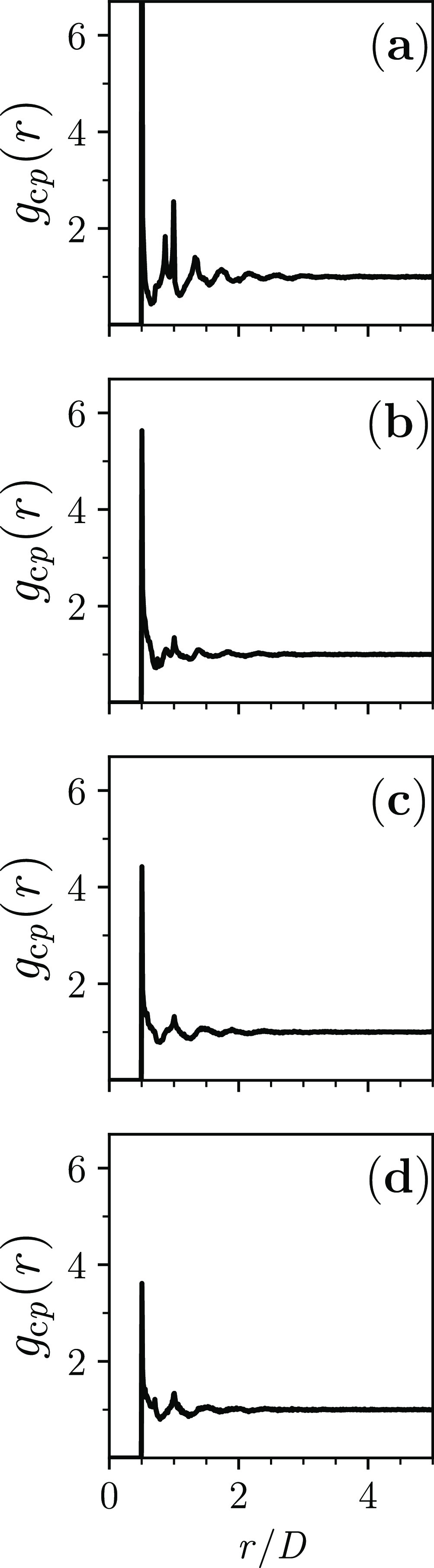
Pair-correlation function *g*_cp_(*r*) for dense disordered jammed packings of hard spherocylinders with (a) *L*/*D* = 0; (b) *L*/*D* = 0.5; (c) *L*/*D* = 1; and (d) *L*/*D* = 1.5 (in each panel, the graph is the average over the respective seven jammed configurations). In panel (a), the present *g*_cp_(*r*) corresponds to the hard-sphere “contacts RDF” that was previously calculated.^56^

It is relevant to investigate the microstructure of the contact points via their pair-correlation function *g*_cp_(*r*).^56^ From the hard-sphere point *L*/*D* = 0, the degree of short-range order, which the number and ordinate value of the various peaks of *g*_cp_(*r*) reflect, decreases with *L*/*D*: *g*_cp_(*r*) evolves to resemble ever more the unit-step function  (Figure 9). This is the form that *g*_cp_(*r*) would have if the contact points were completely uncorrelated. Thus, the form that *g*_cp_(*r*) progressively acquires with *L*/*D* is not inconsistent with the basic assumption of the older random contact equation^60,61^ that aimed to explain the values of ϕ_MRJ_ of hard, very elongated particles. In the derivation of that equation,^60,61^ it was assumed that the contact points become increasingly uncorrelated as the elongation of the hard particles increases.

## 4 Conclusions

This work has numerically generated and studied dense disordered jammed packings of hard spherocylinders with *L*/*D* ∈ [0,2].

On increasing *L*/*D* from the hard-sphere point *L*/*D* = 0, the packing fraction first ascends, then maximizes at *L*/*D* = 0.45 ± 0.05 with a value of 0.721 ± 0.001, and finally descends, while the mean number of contacts per hard spherocylinder first ascends until the isostatic value of 10 is nearly attained at *L*/*D* = 0.45 ± 0.05 and then it is maintained at larger values of *L*/*D*.

The present results agree with previous results on maximally random jammed packings of hard-prolate ellipsoids^10,14^ but partially agree with previous results on dense disordered compact packings of hard spherocylinders.^36−43^ Distinctly from these, the present results point out the isostaticity of the maximally random jammed packings of hard sufficiently elongated spherocylinders and the considerable, rather than negligible, role that the extremal hard hemispheres have in establishing contacts between hard spherocylinders with *L*/*D* ≤ 2.

For hard spherocylinders with *L*/*D* > 2, more numerical simulations are required to accurately determine not only the packing fraction of the maximally random jammed packings but also the mean number of contacts per hard spherocylinder and their type. If these numerical simulations are conducted for hard spherocylinders with *L*/*D* ≫2, they will contribute to a complete test of the newer random contact equation^62^ that aims to predict the values of the packing fraction of maximally random jammed packings of hard very elongated particles in terms of the mean number of contacts per hard very elongated particle.

Qualitatively, the rise in the maximally random jammed state packing fraction of hard-nonspherical particles, as the hard-sphere point is departed from, is related to the concurrent rise in the mean number of contacts per hard-nonspherical particle until isostaticity is attained and successively maintained so that excluded-volume effects cause the successive decrease of the maximally random jammed state packing fraction. Quantitatively, this behavior is yet to be fully understood. One theoretical approach that focuses on the slope of the rise of the maximally random jammed state packing fraction at the hard-sphere point for a number of hard-nonspherical particles has been recently proposed, but to date, its predictions remain essentially untested.^63^

One further aspect that may deserve close attention originates from the observation that the value of *L*/*D* at which the plastic(rotator)-crystal phase disappears in the equilibrium phase diagram of hard spherocylinders^34,44^ seemingly coincides with the value of *L*/*D* at which the nonequilibrium maximally random jammed state of hard spherocylinders attains the maximal packing fraction and isostaticity. Similar observations could also be made for hard ellipsoids (cf. their equilibrium phase diagram^64−67^ with the packing fraction and staticity of their nonequilibrium maximally random jammed states^10,14^) and hard convex lens-shaped particles (cf. their equilibrium phase diagram^68^ with the packing fraction and staticity of their nonequilibrium maximally random jammed states^69,70^). It is unclear whether this is a mere coincidence or rather the symptom of anything more profound in the hard-nonspherical-particle (jammed) configuration space.
